# Anesthetic Protocols for Enhancing Physiological Stability in Rabbits During Hemorrhagic Shock

**DOI:** 10.1155/vmi/6645642

**Published:** 2025-10-13

**Authors:** Ştefania-Mădălina Dandea, Cosmin-Petru Peștean, Iulia Melega, Razvan-Andrei Codea, Lucia-Victoria Bel, Alina-Diana Hașaș, Cristian-Paul Popovici, Bogdan Sevastre

**Affiliations:** Faculty of Veterinary Medicine, University of Agricultural Sciences and Veterinary Medicine, Cluj-Napoca, Romania

**Keywords:** anesthesia protocol, constant rate infusion, hemorrhagic shock, physiological stability, rabbits

## Abstract

**Study Background:**

Rabbits are commonly used in experimental research; however, their sensitivity to handling-induced stress and cardiovascular instability poses considerable challenges. These complications are not fully prevented by standard preanesthetic medication, making them less reliable, particularly in studies involving hemorrhagic shock.

**Aim:**

The aim of this study was to identify enhanced anesthetic protocols that effectively maintain physiological stability and prevent respiratory or cardiac failure during the induction of hemorrhagic shock.

**Methods:**

Fifteen adult female New Zealand White rabbits were divided into three equal groups: one received dexmedetomidine and ketamine; the other received the same induction followed by isoflurane maintenance; while the third, in addition to the medication of the second group, received a constant rate ketamine infusion. Vital parameters such as heart rate, blood pressure, oxygen saturation, end-tidal CO_2_, reflexes, and core body temperature were continuously monitored. Hemorrhagic shock was induced by withdrawing 40% of the total blood volume through a surgically cannulated carotid artery.

**Results:**

Among the three, the third protocol provided the most consistent anesthesia depth in conjunction with stable vital signs, ensuring animal safety and effective modeling of hemorrhagic shock.

**Conclusions:**

These results support the use of a combined inhalant anesthetic with continuous ketamine infusion to enhance physiological stability in rabbits during complex procedures, ultimately improving both the reliability of experimental data and animal welfare.

## 1. Introduction

Anesthetic management in rabbits undergoing hemorrhagic shock necessitates careful consideration to ensure both safety and efficacy [1]. A range of anesthetic techniques has been proposed to address the unique challenges inherent in rabbit anesthesia, including stress induced by handling and the risk of apnea during induction with volatile agents. Injectable anesthesia, particularly as a means of induction, is often preferred to mitigate these issues [2]. The combination of ketamine and medetomidine has been established as a viable alternative anesthetic approach for rabbits, demonstrating success in facilitating surgical procedures [3, 4].

Rabbits exhibit significantly higher perioperative mortality rates compared to companion animals such as dogs and cats, with cardiovascular and respiratory complications often being the primary contributors to these fatalities. Understanding the specific anesthetic risk factors in rabbits is essential for mitigating these risks and enhancing survival rates during and after surgical interventions [5].

The administration of general anesthesia induced by a combination of ketamine and medetomidine (Ket-Med) has been demonstrated to be effective for surgical procedures in rabbits [6]. Recommended dosing regimens of Ket-Med, such as 15.0 mg/kg of ketamine combined with 0.25 or 0.5 mg/kg of medetomidine, administered either subcutaneously or intramuscularly, provide safe and efficient anesthesia for procedures such as ovariohysterectomy and orchiectomy, with occasional supplementation by isoflurane. Increasing the dose of medetomidine typically reduces the need for additional anesthetic support [6]. While high doses of α2-adrenergic agonists may increase the risk of myocardial ischemia, particularly in species with limited coronary collateral circulation and under stress, dexmedetomidine—a highly selective α2 receptor agonist—has been shown to exert cardioprotective effects through sympatholytic and anti-inflammatory mechanisms. In a randomized controlled trial, dexmedetomidine administration during cardiac surgery reduced perioperative myocardial injury and provided renal protection [7]. Similarly, an experimental rabbit study demonstrated that dexmedetomidine decreased the no-reflow area of ischemic myocardium, improved cardiac function, and mitigated ischemia-reperfusion injury [8]. These findings support its use as part of a multimodal anesthetic protocol while highlighting the need for cautious dosing to balance potential risks and benefits.

To minimize stress during the procedure, it is suggested that medetomidine be administered initially, followed by the delivery of oxygen via a mask, and then ketamine [6, 9]. Subcutaneous administration is generally better tolerated than intramuscular injection and facilitates a more rapid loss of consciousness [6, 10]. For enhanced anesthesia in prolonged procedures, the use of sevoflurane, isoflurane, or local anesthetics can be incorporated. Should additional anesthesia be necessary, a diluted dose of ketamine and medetomidine can be administered intravenously, with partial reversal achievable through the use of atipamezole if needed [11, 12].

Anesthesia in hemorrhagic shock models presents unique challenges, as it must ensure the requisite depth of anesthesia while preventing undue depression of circulatory function. Therefore, the aim of our study was to identify optimized anesthetic protocols that not only maintain physiological stability but also prevent cardiac failure during the induction of hemorrhagic shock, all while ensuring sufficient anesthetic depth.

## 2. Materials and Methods

### 2.1. Animals

The study was conducted using fifteen female New Zealand White rabbits, with body weights of 4.13 ± 0.24 kg. During the study design phase, online tools such as Sample Size Calculators and Power and Sample Size | Free Online Calculators were used to ensure that our design was aligned with statistical validity and ethical acceptability. The proposed group size was also approved by the institutional ethics committee, balancing scientific relevance with the imperative to reduce animal use in line with the 3Rs principles. The animals were housed under standard conditions, allowed a 2-week acclimatization period, and were fed a standard diet (“Cantacuzino” National Institute for Medical-Military Research and Development, Bucuresti, Romania). Water and sterilized hay/straw mixtures were freely available. Then, they were divided into three equal experimental groups, each receiving a distinct anesthetic protocol. At the end of the study, all animals were humanely euthanized while they were still unconscious, by anesthetic overdose.

### 2.2. Bioethics

The experimental protocol followed the ARRIVE protocol guidelines; the study complies with Directive 63/2010/EU. According to Romanian National Law 43/2014, the project was approved by the Institutional Ethics Committee (Decision no. 290/22.11.2021) and authorized by the Cluj Sanitary-Veterinary and Food Safety Department (Authorization no. 284/21.12.2021). Highly trained professionals performed all procedures, including animal preparation steps such as trimming, catheter placement, and handling, and conducted them under general anesthesia to minimize stress and ensure animal welfare. The severity of the study was classified as nonrecovery.

### 2.3. Anesthetic Protocols

Animals were divided into three equal groups (5 animals/group). In the dexmedetomidine–ketamine protocol (Group 1), anesthesia was administered using dexmedetomidine and ketamine only. The dexmedetomidine–ketamine–isoflurane protocol (Group 2) included combining dexmedetomidine and ketamine, with the administration of isoflurane 1.5% through an endotracheal (ET) tube. The last anesthesia protocol—dexmedetomidine–ketamine–isoflurane–ketamine cri (Group 3) used dexmedetomidine and ketamine, followed by administration of isoflurane through an endotracheal tube and continuous infusion of ketamine at a rate of 2 mg/kg/h [13].

To ensure adequate analgesia, all rabbits received premedication consisting of intramuscular administration of buprenorphine at a dose of 0.04 mg/kg and subcutaneous administration of meloxicam at a dose of 0.5 mg/kg. Buprenorphine was primarily included for its potent analgesic properties; however, it is worth noting that at doses of 0.01–0.05 mg/kg, it may also provide mild sedation in rabbits, particularly when administered 30–60 min prior to surgical procedures [14, 15]. In this study, the main sedative effects were achieved through the combined administration of dexmedetomidine and ketamine, while buprenorphine served primarily as an analgesic adjunct. Before anesthesia induction, animals were preoxygenated for 5 min. Once premedication had achieved the desired effect, anesthesia induction was performed on all rabbits involved in the study. Induction was achieved by intramuscular administration of dexmedetomidine at a dose of 0.08 mg/kg and ketamine at a dose of 40 mg/kg [16]. The choice of this induction protocol aimed to achieve the desired depth of anesthesia for subsequent procedures.

Following the medication administration, rabbits were prepared for subsequent procedures. This included oxygen supplementation via a facial mask. After these procedures, anesthesia maintenance was performed using isoflurane via endotracheal tubes with diameters ranging from 2 to 3.5 mm for groups 2 and 3, while for Group 1, only oxygen supplementation was used via an ET tube. A rigid endoscope (Eickemeyer Veterinary Equipment, Tuttlingen, Germany, Rigid endoscope 2.7 mm, LED) with a light source was used to guide the intubation process. This was followed by preparation of the auricular regions by shaving them to allow vascular access to the auricular veins. After intubation, all previously shaved regions underwent antisepsis using alcohol (70%) and chlorhexidine (0.05%) solutions. To ensure venous access, the left lateral auricular and cephalic veins were catheterized using a 22-G venous catheter. This catheterization procedure allowed for reliable and convenient venous access for the administration of fluids or medications during the anesthesia process.

The ventral region of the neck and proximal thorax were also shaved to facilitate surgical access. Additionally, hair on the limb extremities was trimmed, ECG electrodes were placed, and experimental models were positioned in dorsal recumbency and fixed in the chosen position using adhesive strips.

Throughout these procedures, reflex responses were carefully monitored to assess the depth of anesthesia every 15 min. Reflexes were clinically assessed by pressing the ear and checking the pedal reflex on the right hind limb, palpebral reflexes, and ear pinch reaction, during vitals' monitoring. A positive reflex was noted, even if there was an isolated time of the registered reflex. Negative reactions were recorded if evaluated reflexes were absent throughout the whole length of the experimental procedures.

Within the third protocol of the study, a syringe pump with a constant rate infusion of ketamine was connected using an Eickemeyer InjectoVET Easy II injector (Eickemeyer Veterinary Equipment, Tuttlingen, Germany; infusion rate range: 0.1–1.500 mL/h; accuracy ± 2%) to provide a controlled and desired infusion rate during the experimental protocol. The calculated volume of ketamine was diluted with sterile saline in a 10 mL syringe and administered intravenously at a constant rate of 5 mL/h using the syringe pump; no additional fluid therapy was provided during the procedures. The rabbits' temperature was monitored by placing an esophageal tube, and the readings were displayed on the main monitor. All individuals in the considered sample were placed on an electric pad to maintain physiological body temperature throughout the procedures.

### 2.4. Hemorrhagic Shock Induction

**Figure 1:**
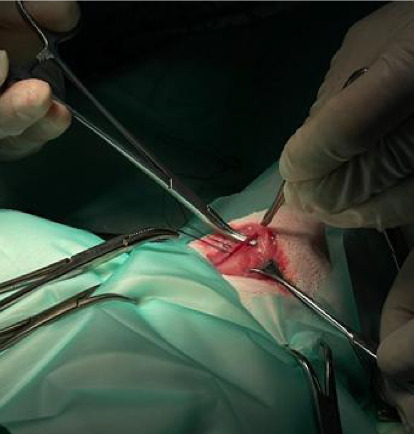
Surgical exposure of the carotid artery-dorsal exposure of the carotid arteries was achieved using subcutaneous sutures (3-0) secured in Adson hemostatic forceps.

**Figure 2:**
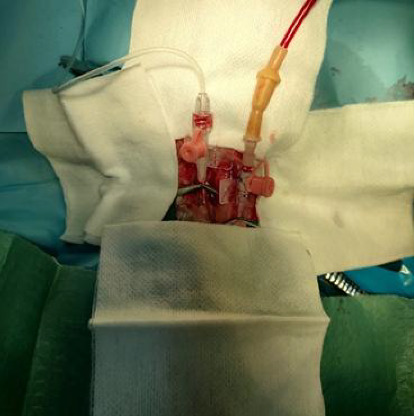
The right carotid artery was catheterized with a sterile 22-G venous catheter for the invasive blood pressure monitoring transducer; the left carotid artery was catheterized with another 22-G venous catheter to serve as a bleeding site.

All rabbits underwent a controlled induction of hemorrhagic shock. After performing preoperative procedures and ensuring the antisepsis of the cervical region, an incision was made along the ventral midline of the neck, approximately 3 cm in length, to expose the underlying tissues. The cervical muscles and surrounding anatomical structures were dissected to expose both carotid arteries. For better exposure and visualization of the procedural area, Desmarres or Knapp retractors were used to retract the cervical muscles and surrounding anatomical structures. Dorsal exposure of the carotid arteries was achieved using subcutaneous sutures (3-0) secured in Adson hemostatic forceps (Figure 1). The right carotid artery was catheterized with a sterile 22-G venous catheter and connected to an invasive blood pressure Okamo [17] monitoring transducer to provide precise data on mean arterial pressure (MAP), systolic pressure, and diastolic pressure. The left carotid artery was catheterized with another 22-G venous catheter to serve as a bleeding site (Figure 2).

**Figure 3:**
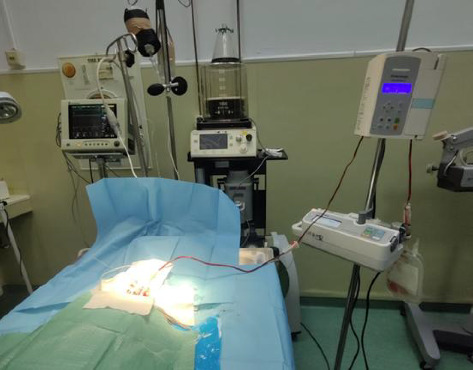
The catheter inserted into the right carotid artery was connected to a transfusion tube leading to a transfusion bag.

Approximately 40% of the total blood volume was steadily extracted over 1 h. To maintain constant and controlled bleeding, the bleeding tube was connected to an Eickemeyer InfusoVet II infusion pump with the tubing inverted (Figure 3) and finally collected and stored in a 250-mL Demotek single blood transfusion bag (Demophorius Healthcare Ltd., product code BBS; prefilled with CPDA-1 anticoagulant; transparent PVC construction). This configuration ensured controlled exsanguination. The total volume of blood extraction was set as the infusion volume on the machine at the same infusion rate. After stopping the bleeding, a monitoring period of 30 min was allowed to observe the evolution of hemorrhagic shock signs.

### 2.5. Duration and Monitored Parameters

The total duration of the experiment was 120 min, during which vital signs and reflexes were continuously monitored. Monitoring was performed using a veterinary multiparameter monitor (PM 9000Vet, Mindray Bio-Medical Electronics Co., Shenzhen, China), which continuously recorded the heart rate, respiratory rate, SpO_2_, ETCO_2_, systolic blood pressure, diastolic blood pressure, mean arterial pressure, and temperature via an esophageal probe. End-tidal CO2 (ETCO_2_) levels were measured using a transducer connected to the monitor. All monitored aspects were observed at different times during the procedure: before surgical procedures, after preoperative preparation, noted as T0 in the results. After 30 min, during which surgical procedures were performed, and arteries were catheterized, the bleeding time began, noted as T1, then at every 15 min during the bleeding time, noted as T2 (45 min from the start of the experiment), T3 (60 min), T4 (75 min), and T5 (90 min). At T5, bleeding was stopped, and additional data were observed after 30 min at T6, totaling 120 min from the start of the experiment.

At the end of the protocol, animals were humanely euthanized by isoflurane overdose in accordance with current euthanasia in accordance with current AVMA and UMB Guidelines [18]. Death was confirmed by the absence of heart sounds on auscultation, absence of respiratory movements, lack of corneal and pedal reflexes, and continuous monitoring which showed a drop in blood pressure and an isoelectric EGC signal.

### 2.6. Statistical Analysis

The data were expressed as the mean ± standard deviation (SD), and the normal distribution of the data was assessed using the Shapiro–Wilk test. Statistical evaluation was conducted using the multivariate analysis of variance (ANOVA two-way) test, followed by Bonferroni post-test, and results were considered statistically significant at a *p* value of < 0.05.

The data analysis and graphic generation were done using GraphPad Prism 5.0 for Windows, GraphPad Software, San Diego, California.

## 3. Results and Discussion

The study investigated vital parameters during anesthesia in rabbits undergoing controlled hemorrhagic shock. It assessed the effects of various anesthetic protocols on the stabilization of these parameters, the depth of reflex responses, and the progression of signs associated with hemorrhagic shock.

### 3.1. Comparative Invasive Measurements of Arterial Blood Pressure

The results of invasive blood pressure monitoring provide valuable insights into the effects of anesthesia on cardiovascular and hemodynamic status during the induction of hemorrhagic shock. These findings offer a basis for optimizing anesthesia protocols to ensure hemodynamic stability in experimental models.

**Figure 4:**
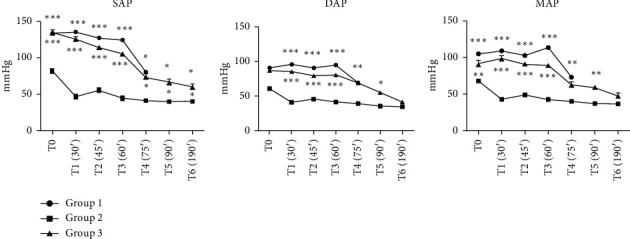
Arterial blood pressure parameters during experimental procedures (mean ± SD, 5 animals/group), ^∗^*p* < 0.05, ^∗∗^*p* < 0.01, ^∗∗∗^*p* < 0.001, as compared with Group 2; SAP = systolic arterial pressure. DAP = diastolic arterial pressure. MAP = mean arterial pressure. Group 1-dexmedetomidine and ketamine, Group 2- dexmedetomidine and ketamine, with the administration of isoflurane at rates of 1.5%–2%, Group 3: dexmedetomidine and ketamine, followed by administration of isoflurane and continuous infusion of ketamine. Reference range in SAP = 90–130 mmHg, reference range DAP = 80–90 mmHg. Reference range MAP = 83–103 mmHg, T0: before surgical procedures after preoperative preparation; T1 = after 30 min, carotid arteries catheterized, bleeding time started, then every 15 min during bleeding time-T2 (45 min from the beginning of the experiment), T3 (60 min), T4 (75 min), and T5 (90 min). At T5, bleeding stopped and additional data were observed after 30 min at T6.

As shown in Figure 4, Group 1 (dexmedetomidine and ketamine) exhibited a gradual decline in systolic, diastolic, and mean arterial pressures. However, the depth of anesthesia in Group 1 was deemed insufficient, as evidenced by the presence of positive reflexes and changes in the respiratory rate at T5. In response, additional intravenous ketamine infusions were administered, though scientific relevance was lost due to the variability in the data. Group 2, while demonstrating better depth of anesthesia control, experienced significant drops in blood pressure, likely attributable to the vasodilatory effects of isoflurane. This resulted in complications during vascular procedures, such as catheter placement and fixation, thus prolonging and complicating the overall experimental procedure. In contrast, Group 3 (continuous ketamine infusion) exhibited a more stable blood pressure trend with significant statistical differences (*p* < 0.001) in comparison with Group 2. The blood pressure parameters indicate that the anesthesia protocol had a significant impact on blood pressure dynamics during hemorrhagic shock induction. Group 3, which included continuous ketamine infusion, maintained a relatively stable blood pressure profile compared to the other groups.

Likewise, previous studies indicate that volatile anesthetics were associated with a dose-dependent decline in blood pressure, its extent varying among protocols and various volatile anesthetics [19]. Furthermore, in the isoflurane-induced anesthesia, the dose-dependent cardiopulmonary depression is likely associated with the vasodilatory effects and negative inotropic properties [20].

#### 3.1.1. Vital Signs

**Figure 5:**
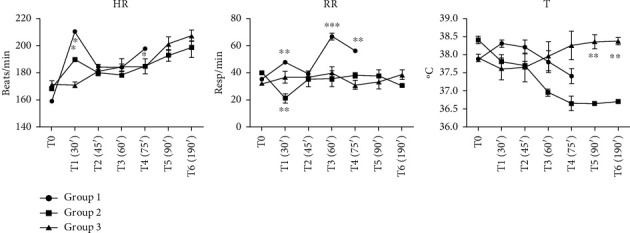
Heart rates, respiratory rates, and core body temperatures during the experimental procedure (mean ± SD, 5 animals/group) ^∗^*p* < 0.05, ^∗∗^*p* < 0.01, -^∗∗∗^*p* < 0.001, as compared with Group 2; HR = heart rate; RR = respiratory rate. T = core body temperature. Group 1: dexmedetomidine and ketamine, Group 2: dexmedetomidine and ketamine, with the administration of isoflurane at rates of 1.5%–2%, Group 3: dexmedetomidine and ketamine, followed by administration of isoflurane and continuous infusion of ketamine. Reference range in HR = 130–325 (/min); RR = 30–60 (/min); T = 38.5–40 (°C), T0: before surgical procedures after preoperative preparation; T1 = after 30 min, carotid arteries catheterized, bleeding time started, then every 15 min during bleeding time, T2 (45 min from the beginning of the experiment), T3 (60 min), T4 (75 min), and T5 (90 min). At T5, bleeding stopped and additional data were observed after 30 min at T6.

In Group 1 (Figure 5), which received anesthesia with dexmedetomidine and ketamine only, the heart rate at the onset of the experiment (T0) was approximately 159 bpm, with subsequent values generally remaining within the reference range for most measurements. At T1 (30 min), however, a significant increase was observed, with heart rate rising to 210 bpm. This increase was accompanied by tachypnea and could be interpreted as a sign of insufficient anesthesia depth. In contrast, in Groups 2 and 3, which included additional anesthetic agents, the heart rate maintained a rather stable evolution. A potential risk associated with anesthesia in rabbits is bradycardia, where heart rates below 65 bpm may pose life-threatening risks [21]. Bradycardia is expected under anesthesia due to increased vagal tone and certain drug effects; however, such values were absent in the present study, consistent with the cardiovascular stability typically reported under dexmedetomidine–ketamine protocols [4]. On the other hand, an elevated heart rate can indicate pain or insufficient analgesia [22], and in Group 1, it was observed together with tachypnea, suggesting a potential insufficient anesthetic depth in the absence of isoflurane. At the same time, tachycardia is also considered a normal physiological response during the induction of hemorrhagic shock [6], reflecting sympathetic activation.

In terms of respiratory dynamics, tachypnea was also noted in Group 1. The occurrence of tachycardia and tachypnea could be interpreted as a sign of insufficient anesthesia depth, considering that volatile anesthetic supplementation was not part of this protocol. In Groups 2 and 3, only minor variations in the respiratory rate were observed, and all values remained within the reference range, indicating stable respiratory function throughout the experimental period. Group 2, which underwent inhalational anesthesia with isoflurane, displayed lower respiratory rates (17.5 to 35 breaths per minute). It is critical to ensure appropriate anesthetic depth in order to avoid adverse reactions and safeguard the well-being of rabbits during anesthesia. However, if present, tachypnea can be attributable to compensatory mechanisms associated with hemorrhagic shock, as the reduction in circulating blood volume and oxygen delivery triggers an increase in respiratory drive in an attempt to maintain tissue oxygenation and compensate for metabolic acidosis [23].

In Group 1, body temperature ranged from 37.35°C to 38.3°C, generally within the reference range, though closer to the lower limit. Group 2 exhibited lower temperatures, either within or below the reference range. In Group 3, temperatures largely remained within the reference range, hovering around the lower physiological limits. The use of an electric heating pad throughout anesthesia underscores the hypothermic effect induced by the anesthetic. This phenomenon is well documented, with general anesthesia typically resulting in moderate temperature reductions due to anesthesia-induced vasodilation and the depression of hypothalamic thermoregulatory centers [24].

Mild hypothermia is a common occurrence during anesthesia and surgical procedures, primarily due to anesthetic-induced vasodilation and redistribution of core body heat to the periphery. Volatile anesthetics are particularly associated with impaired thermoregulation and heat loss. In contrast, ketamine has been reported to have less pronounced vasodilatory effects and may help preserve thermoregulation, thereby reducing the risk of hypothermia during anesthesia [25]. In our study, body temperature was maintained near 38°C in Group 3, suggesting that the anesthetic protocol—particularly the inclusion of ketamine cri—may have contributed to limiting perioperative hypothermia and preventing further interference with the pathophysiological responses to hemorrhagic shock.

### 3.2. Respiratory Gases and Oxygen Saturation

**Figure 6:**
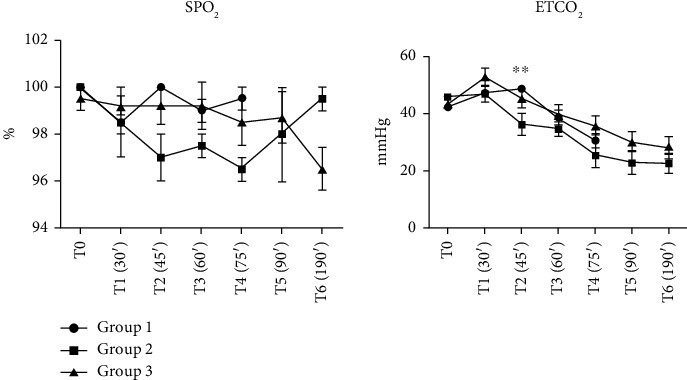
Respiratory gases and oxygen saturation at various time points during experimental procedures (mean ± SD, 5 animals/group), ^∗∗^*p* < 0.01 as compared with Group 2; HR; % = oxygen saturation. ETCO_2_ = end-tidal carbon dioxide in expiratory air. Group 1: dexmedetomidine and ketamine, Group 2: dexmedetomidine and ketamine, with the administration of isoflurane at rates of 1.5%–2% Group 3: dexmedetomidine and ketamine, followed by administration of isoflurane and continuous infusion of ketamine. Reference range in SO_2_% = 96–100%. Reference range ETCO_2_ = 35–45 mmHg, T0: before surgical procedures after preoperative preparation; T1 = after 30 min, carotid arteries catheterized, bleeding time started, then every 15 min during bleeding time- T2 (45 min from the beginning of the experiment), T3 (60 min), T4 (75 min), and T5 (90 min). At T5, bleeding stopped and additional data were observed after 30 min at T6.

Throughout the experimental procedure, oxygen saturation (SpO_2_) remained within the physiological reference range (96%–100%) across all groups, indicating adequate oxygenation despite the experimental manipulations (Figure 6). Initially, end-tidal CO_2_ (ETCO_2_) showed an upward trend; however, from T4 onward, ETCO_2_ levels fell below the physiological range in all groups, with the most pronounced reductions observed in Group 2. By T6, the ETCO_2_ values were 23.5 ± 2.5, 22.5 ± 3.5, and 28.0 ± 3.8 mmHg in Groups 1, 2, and 3, respectively. This pattern suggests the development of hypocapnia, potentially linked to hyperventilation, reduced perfusion, or metabolic alterations resulting from the experimental conditions, particularly during the hemorrhagic phase. The relatively higher ETCO_2_ levels observed in Group 3 at later time points may suggest a more stable cardiopulmonary status or a distinct compensatory response to the hemorrhagic challenge. However, it is also important to consider the potential influence of apparatus dead space. A reinhalation circuit system was employed in this study, and in small animals such as rabbits, the presence of dead space within the breathing circuit can lead to partial CO_2_ rebreathing—particularly when respiratory rates are reduced. As such, elevated ETCO_2_ values may not solely reflect physiological compensation or perfusion status but could also be influenced by system-related factors.

Yao et al. suggested that throughout the progression of hemorrhagic shock, impaired lung perfusion and reduced cardiac output lead to increased anaerobic metabolism, which in turn, alters ETCO_2_ levels [26]. Consequently, ETCO_2_ could serve as a valuable, noninvasive indicator of microcirculatory function and could contribute to the early identification of hemorrhagic shock. However, elevated inspiratory CO_2_ concentrations are caused by a reduced respiratory rate in animals receiving dexmedetomidine; increasing the dose of medetomidine primarily led to a rise in end-tidal CO_2_ levels during anesthesia, with minimal impact on cardiovascular depression [6, 27, 28].

These findings emphasize the importance of interpreting ETCO_2_ values in the context of the anesthetic protocol, breathing system design, and respiratory pattern. While ETCO_2_ remains a valuable, noninvasive indicator of pulmonary perfusion and ventilatory adequacy, it should be carefully correlated with other physiological parameters, such as SpO_2_ and respiratory rate, and potentially confirmed by arterial blood gasometry to avoid misinterpretation—especially during states of hemodynamic compromise.

### 3.3. Depth of Anesthesia

Anesthetic depth was assessed through reflex responses, including the ear pinch, pedal withdrawal, and palpebral reflexes, which were absent in all groups within 10–15 min following induction. However, Group 1 exhibited a positive pedal reflex at T5, indicating insufficient anesthetic depth, which was further manifested by an increase in the respiratory rate. In response, additional intravenous ketamine supplementation was administered to Group 1 animals as a human end-point protocol; however, subsequent data on blood pressure and vital parameters were excluded from statistical analysis due to experimental protocol deviation. Schmid et al. reported that intramuscular anesthetic combinations can provide effective and surgically tolerable anesthesia for short procedures [29]. In contrast, our study required prolonged anesthesia to support the induction and monitoring of hemorrhagic shock. Under these conditions, Groups 2 and 3, which received continuous anesthetic administration after induction, maintained adequate anesthetic depth throughout the experimental timeline, with no return of reflexes. These observations are consistent with our previous studies, which similarly emphasized that maintaining a prolonged, stable, and sufficiently deep plane of anesthesia during the induction of hemorrhagic shock is essential for ensuring the reliability of experimental outcomes [30].

### 3.4. Study Limitations

This study has several limitations that should be considered. First, the small number of animals, dictated by ethical regulations and institutional approval, limits the statistical power and may reduce the generalizability of the results. Second, the focus was restricted to short-term monitoring during anesthesia and the induction of hemorrhagic shock, which allowed evaluation of immediate physiological and anesthetic responses but did not account for potential long-term effects such as delayed cardiovascular, metabolic, or neurological complications. Third, the exclusive use of healthy adult rabbits may not fully replicate the variability seen in clinical or diseased populations. Despite these limitations, the study offers valuable insight into optimizing anesthetic protocols for physiological stability during invasive shock models and serves as a foundation for future research involving prolonged observation, broader anesthetic comparisons, and diverse animal models.

## 4. Conclusions

This study identified a potential anesthetic protocol for extended experimental procedures involving hemorrhagic shock in rabbits. While intramuscular combinations, such as dexmedetomidine–ketamine, provided effective anesthesia for short durations, without maintenance, their depth diminished over time. Dexmedetomidine–ketamine group (Group 1) exhibited inadequate anesthesia and required additional ketamine supplementation. Protocols that combined dexmedetomidine–ketamine induction with isoflurane maintenance (Groups 2 and 3) offered improved anesthetic stability, with Group 3—featuring a continuous ketamine infusion, demonstrating adequate anesthesia depth and control over vital parameters. Although isoflurane contributes to stable anesthesia, its vasodilatory effects can complicate surgical access to vascular structures, such as the carotid artery, making catheter placement more challenging. In this regard, the Group 3 protocol, which combines dexmedetomidine–ketamine induction, isoflurane maintenance, and continuous ketamine infusion, provided the best balance between anesthetic depth, physiological stability, and surgical feasibility. These findings support its use as a reliable and welfare-conscious approach for extended anesthetic procedures in hemorrhagic shock models in rabbits.

## Data Availability

All original data are available from the corresponding author.
